# Genome-wide identification and expression profiling of bHLH transcription factors associated with ferulic acid biosynthesis in *Angelica sinensis*

**DOI:** 10.3389/fpls.2025.1718585

**Published:** 2025-11-26

**Authors:** Khadija Tehseen Arshad, Chaohui Li, Lesong Li, Juan Wang, Junwen Chen, Yan Zhao

**Affiliations:** 1 Key Laboratory of Medicinal Plant Biology of Yunnan Province, National & Local Joint Engineering Research Center on Germplasms Innovation & Utilization of Chinese Medicinal Materials in Southwest China, Yunnan Agricultural University, Kunming, China; 2 College of Agronomy & Biotechnology, Yunnan Agricultural University, Kunming, China; 3 Yunnan Characteristic Plant Extraction Laboratory, Kunming, Yunnan, China

**Keywords:** *Angelica sinensis*, bHLH transcriptionfactors, whole-genome identification, bioinformatics analysis, gene expression analysis

## Abstract

This study identifies 148 *bHLH* transcription factors in *Angelica sinensis* and reveals four putative candidates associated with ferulic acid biosynthesis, providing a genetic foundation for metabolic engineering to enhance the plant’s medicinal value. The basic helix-loop-helix (bHLH) proteins regulate plant growth, development, stress responses, and secondary metabolites. While well-characterized in woody plants, they remain unexplored in *Angelica sinensis* (*A. sinensis*), a medicinal plant renowned for bioactive compounds including ferulic acid. Therefore, we systematically identified and characterized *bHLH* transcription factors in *A. sinensis* through whole-genome analysis and transcriptome profiling, identifying putative genes potentially regulating ferulic acid biosynthesis. Bioinformatic analyses were employed to characterize the physicochemical properties, gene structures, conserved motifs/domains, phylogenetic relationships, chromosome localization, collinearity, cis-acting elements, and transcriptome expression patterns of *AsbHLHs*. A total of 148 *AsbHLH* genes were annotated from the genomic database of *A. sinensis*, classified into 16 subfamilies based on the phylogenetic analysis. Results revealed that these transcription factors encode hydrophilic proteins (83–741 aa; 9.6–80.8 kDa), with nearly all localized to the nucleus. Gene structure analysis showed exon numbers ranging from 1 to 18, while MEME motif analysis identified five conserved motifs (1–5) shared across most *AsbHLH* proteins. Promoter analysis uncovered abundant *cis*-elements associated with growth, secondary metabolism (including ferulic acid biosynthesis), and stress responses. WGCNA revealed turquoise module contained 40 *bHLH* and five phenylpropanoid pathway-specific genes, from which PPI and phylogenetic analyses pinpointed four putative genes potentially associated with ferulic acid production. Quantitative RT-PCR validated these candidates, showing expression patterns consistent with transcriptome data. This study provides the first comprehensive genomic/transcriptomic resource for *AsbHLH* genes in *A. sinensis*, highlighting their secondary metabolic roles. Identified candidates enable genetic engineering strategies to boost ferulic acid production, enhancing *A. sinensis’* medicinal value.

## Introduction

A perennial herbaceous plant, *Angelica sinensis* (Oliv.) Diels belongs to the Apiaceae family, has been broadly used in traditional medicines due to the therapeutic properties of its dried root, which include blood nourishment, triggering blood circulation, regulating menstruation, relieving pain, and endorsing intestinal motility (Zhang et al., 2012; Shedoeva et al., 2019; Yuan et al., 2019; Guo et al., 2020; Chen et al., 2021; Han et al., 2023; Tian et al., 2024). These pharmacological properties are mainly attributed to bioactive compounds such as phenolic acids, flavonoids, coumarins, polysaccharides, and phthalides (Sarker and Nahar, 2004; Batiha et al., 2022; Sabeel et al., 2023; Wang et al., 2023; Mishra and Paliwal, 2025; Ren et al., 2025; Zhao et al., 2025). Recent studies have primarily focused on the chemical composition (Chen et al., 2022; Sun et al., 2024), pharmacological effects (Kim et al., 2023; Hu et al., 2024), and biological activities (Lee et al., 2023; Wang et al., 2023) of *A. sinensis*, while the systematic research on germplasm quality improvement remains limited. Therefore, there is an urgent need to advance breeding programs for quality improvement and to explore the putative regulatory genes associated with the biosynthesis of its secondary metabolites.

The genome-wide study is foundational for explicating gene families and their regulatory networks, and the reliability of such analysis depends upon the quality and wholeness of the reference genomic resource used. For *Angelica sinensis*, the genomic database has been pointedly improved in recent years. Subsequently, the initial draft genomes and several high-quality, chromosome-level assemblies have now been published (Han et al., 2022; Li et al., 2023; Dang et al., 2024; Wu et al., 2024). These databases provide complementary tools for the research community. It is extensively documented that exploiting the most contiguous and highly annotated assembly is the finest practice for exploiting the extensiveness of gene family identification (Dang et al., 2024; Wu et al., 2024). The existence of multiple genomes also suggests a unique chance for cross-validation of findings, confirming strength and correctness.

Ferulic acid is a major phenolic compound plentiful in Chinese herbal medicines, plays a key structural role in plant cell walls by esterifying with polysaccharides and facilitating cross-linking between lignin and hemicellulose (Mnich et al., 2020; Robert et al., 2022). In *A. sinensis*, ferulic acid is a principal bioactive compound (Giacomelli et al., 2017; Dong et al., 2022), contributing significantly to its traditional practices, particularly in treating gynecological disorders (Jiao et al., 2022; Pei et al., 2024). Its therapeutic benefits, especially improved blood circulation, menstrual regulation, and pain relief, have been largely attributed to its presence in the plant (Cheng et al., 2012; Li et al., 2020; Batiha et al., 2022; Yang X. et al., 2023; Shen et al., 2025). Current research has further tinted ferulic acid’s role in oxidative stress defense and its potential in averting chronic diseases such as cardiovascular disorders, diabetes, and cancer, making it valuable for clinical applications, including anti-inflammatory, anti-carcinogenic, and cardioprotective effects (Kiokias et al., 2020; Li et al., 2021; Afnan et al., 2022; Ma et al., 2022; Pandi et al., 2022; Bao et al., 2023).

At the transcriptional level, several compounds regarding the phenylpropanoid pathway, including ferulic acid biosynthesis, are primarily regulated by “*bHLH* (basic helix-loop-helix)” transcription factor (TF) (Zhao et al., 2023; Gao et al., 2024; Chen et al., 2024). The *bHLH* gene family signifies one of the largest TF families in plants, tangled in diverse biological processes including growth, development, and stress responses (Ke et al., 2020; Hao et al., 2021; Thoben and Pucker, 2023; Zuo et al., 2023; Zhang et al., 2025). These proteins contain a conserved bHLH domain of around 60 amino acids that simplifies DNA binding, classically identifying E-box motifs (CANNTG) in promoter regions (Yesudhas et al., 2017; de Martin et al., 2021; Guo et al., 2021; Zhang et al., 2025). Functionally, *bHLH* TFs regulate several physiological processes through homo- or heterodimer establishment, including hormone signaling, stress adaptation, and secondary metabolism (Feller et al., 2011; Goossens et al., 2016; Meraj et al., 2020; Jan et al., 2021; Khan et al., 2025). Numerous studies have predominantly focused on their role in secondary metabolism, particularly in the biosynthesis of phenylpropanoids such as flavonoids, ferulic acid, and lignins, which are necessary for plant defense mechanisms (Wang et al., 2018; Sharma et al., 2019; Guo et al., 2021; Hussain and Reigosa, 2021; Jan et al., 2021; Rabeh et al., 2025). For instance, *MYC*, a well-characterized *bHLH* TF, regulates the jasmonate signaling pathway essential for plant defense against herbivores and pathogens (Niu et al., 2011; Nakata et al., 2013; Du et al., 2018; Wei et al., 2022; Jia et al., 2025; Kim et al., 2025; Liu et al., 2025), while other *bHLH* TFs are involved in anthocyanin biosynthesis, iron homeostasis, and light-mediated growth (Li et al., 2020; Buti et al., 2020; Jing and Lin, 2020; Qian et al., 2021; Hao et al., 2021; Liu et al., 2021; LaFountain and Yuan, 2021).

Despite the long history of medicinal use of *A. sinensis*, the regulatory mechanisms underlying the biosynthesis of key compounds like ferulic acid remain poorly understood. Ferulic acid is synthesized via the phenylpropanoid pathway (Dong et al., 2022; Guo et al., 2023), yet the specific roles of *bHLH* TFs in modulating this pathway are unclear. Therefore, elucidating these potential regulatory mechanisms is crucial for enhancing the therapeutic potential of *A. sinensis*. Given the importance of ferulic acid and the central role of *bHLH* TFs in secondary metabolism, this study aims to conduct a genome-wide identification of *bHLH* transcription factors in *A. sinensis* to identify putative regulators associated with ferulic acid biosynthesis. To attain this, we used the chromosome-level genome assembly of *Angelica sinensis* (female ginseng) from Han et al., 2022; a resource selected for its inclusive annotation and established value in prior gene family studies. Based on this reference genome, we screened and identified *bHLH* transcription factors, followed by comprehensive bioinformatics analyses including protein physicochemical property assessment, phylogenetic and evolutionary analysis, gene structure, domains, and conserved motif identification, chromosomal localization, synteny analysis, and cis-acting element prediction. By using transcriptome data, gene expression patterns were further investigated, and qRT-PCR was performed to screen out *bHLH* TFs potentially associated with ferulic acid biosynthesis in *A. sinensis*. These efforts will provide a theoretical foundation for future studies on the molecular mechanisms of putative bHLH-regulated ferulic acid biosynthesis and support quality breeding programs for *A. sinensis*.

## Materials and methods

### Plant materials

The genomic sequence and GFF annotation file of *A. sinensis* were derived from the PlantGIR Genomic Database (http://plantgir.cn/Download) (Han et al., 2022). Genome-derived protein sequences were generated with GFFread (v0.12.7), followed by isolation of the longest isoform per gene via a Perl-based filtering script. The reference genome and gene family categorization data for *A. thaliana* were acquired from the TAIR public repository (https://www.arabidopsis.org/) (Swarbreck et al., 2007). The transcriptome data of *A. sinensis* from different tissues (root, leaf and stem) were sourced from our previous study (GSA: CRA016571), publicly accessible in the Genome Sequence Archive (GSA) at the CNCB-NGDC (https://ngdc.cncb.ac.cn/gsa) (Arshad et al., 2024).

### Identification of *bHLH* family genes in *A. sinensis*

The *bHLH* transcription factor family genes in *A. sinensis* were identified through a comprehensive bioinformatics pipeline using the Pfam database (http://pfam.xfam.org/search) for domain prediction (PF00010) and TBtools (v2.096) for initial screening. We obtained reference sequences (225 A*. thaliana* bHLH proteins) from PlantTFDB (https://planttfdb.gao-lab.org/) and performed HMMER (v3.3.1) searches against the *A. sinensis* genome with sequential E-value thresholds (1×10^-5^ followed by 0.001), including domain alignment with ClustalW (v2.1) and HMM profile refinement (Finn et al., 2011). Putative genes were validated through BLAST comparison with *A. thaliana* sequences, SMART database screening for SANT domains, and NCBI-CDD verification (E-value ≤ 0.01), with final identification based on the intersection of HMMER and BLAST results to ensure both domain presence and evolutionary conservation.

### Prediction of physicochemical properties, secondary structure, and subcellular localization of *Angelica sinensis* bHLH proteins

The physicochemical properties of identified bHLH proteins, including relative molecular weight, isoelectric point (pI), and grand average of hydropathicity (GRAVY), were predicted using ExPASy ProtParam (https://web.expasy.org/protparam/) (Dong et al., 2024). Subcellular localization was inferred with Cell-PLoc 2.0 (http://www.csbio.sjtu.edu.cn/bioinf/Cell-PLoc-2/), and secondary structure composition (α-helices, β-sheets, coils, etc.) was analyzed via SOPMA (http://npsa-pbil.ibcp.fr/cgi-bin/npsa_automat.pl?page=npsa_sopma.html). Furthermore, the amino acid configuration of the bHLH protein was examined using DNAMAN.

### Phylogenetic and evolutionary analysis

Phylogenetic analysis of *AsbHLHs* was performed using MEGA 11.0 (Tamura et al., 2021). The maximum likelihood (ML) tree was constructed with 225 A*. thaliana* bHLH sequences (from PlantTFDB) as reference, employing the VT+R6 model (automatically selected as optimal) and 1000 bootstrap replicates. Branch support values (%) are indicated by circles (proportional to bootstrap values) (Price et al., 2010; Horiike, 2016). The Newick-formatted tree (*.NWK) was visualized and annotated using the iTOL web tool (https://itol.embl.de/). Subfamily classification of *A. sinensis* bHLH TFs was inferred based on their clustering with Arabidopsis orthologs.

### Structural analysis of *AsbHLHs*

The genomic coordinates and exon-intron structures were extracted from the genome’s GFF3 annotation file. TBtools was then used to generate visual representations of gene architectures, with coding sequences (CDS), untranslated regions (UTRs), and introns annotated (Chen and Xia, 2022; Wei et al., 2023; Rizwan et al., 2024).

### Identification and visualization of conserved motifs

Conserved motifs within the *A. sinensis* bHLH protein sequences were identified using MEME Suite (v5.5.2; http://meme-suite.org) with the following parameters: maximum motif count = 10, E-value threshold < 0.01, and default settings for width and repetitions (Bailey et al., 2006). The motif architectures were graphically mapped to gene structures using TBtools (v2.096) to analyze positional conservation.

### Conserved domains analysis

The conserved domains were identified using NCBI’s CD-search tool (Conserved Domain Database; https://www.ncbi.nlm.nih.gov/Structure/bwrpsb/bwrpsb.cgi), with default parameters for domain detection (Zhou et al., 2020). Domain architectures were visualized using TBtools to illustrate structural features such as the bHLH DNA-binding and dimerization regions.

### Cis-acting element analysis in *AsbHLH* promoters

The 2,000 bp promoter regions upstream of transcription start sites (TSS) of *A. sinensis bHLH* genes were extracted using TBtools. Cis-acting regulatory elements were identified through the PlantCARE database (http://bioinformatics.psb.ugent.be/webtools/plantcare/html/) (Lescot, 2002), and their results were statistically analyzed and visualized via **ggplot2** (v3.5.1) in R to highlight element distribution and functional categories (Wickham, 2011; He et al., 2021; Li et al., 2024; Tobin et al., 2025).

### Chromosomal localization and synteny analysis

Chromosomal positions were mapped using genome annotation data visualized with TBtools. Intraspecies synteny analysis was performed by self-aligning *A. sinensis* bHLH protein sequences using the “One Step MCScanX-SuperFast” tool in TBtools, with results displayed through “Advanced Circos” visualization. For interspecies analysis, the *A. thaliana* genome annotation (NCBI) was aligned with *A. sinensis bHLH* genes using the same MCScanX pipeline, with syntenic relationships illustrated via Circos plots (Zhang et al., 2025).

### Expression pattern analysis and qRT-PCR

Gene expression analysis of *A. sinensis* bHLH transcription factors was performed using transcriptome data from our previous study and publicly available raw sequences deposited in the Genome Sequence Archive (GSA: CRA016571) at the National Genomic Data Center (https://ngdc.cncb.ac.cn/gsa). Tissue-specific expression patterns of *bHLH* genes, as well as the phenylpropanoid pathway-specific genes responsible for ferulic acid regulation in *A. sinensis*, were quantified using FPKM values in roots, leaves, and stems, and visualized as clustered heatmaps via TBtools, with color gradients representing expression intensity. Weighted gene co-expression network analysis (WGCNA) was performed to construct gene co-expression networks. The protein interaction analysis of differentially expressed genes is based on the STRING database of known and predicted protein–protein interactions. WGCNA was performed as described previously (Langfelder and Horvath, 2008). Protein-protein interaction (PPI) network was constructed using Cytoscape to analyze the interaction among *bHLH* and phenylpropanoid pathway-specific genes identified in the precise module to classify putative genes regarding ferulic acid biosynthesis. Further, we performed phylogenetic analysis to screen identified putative bHLH proteins potentially associated with ferulic acid biosynthesis. qRT-PCR validation was performed using primers designed with Primer3 (https://primer3.ut.ee/; **Supplementary Table 1**) in 20 μL reactions containing SYBR qPCR SuperMix Plus (10 μL), primers (0.4 μL each), cDNA (1 μL), and ddH_2_O (8.2 μL), with thermal cycling conditions of 95 °C for 30 s; 40 cycles of 95 °C for 10 s and 60 °C for 20 s; followed by melt curve analysis. For the calculation of specific gene expression differences, the previously reported ACTIN gene was used as an internal reference gene (Arshad et al., 2024). Relative expression (2^-ΔΔCt^ method) was calculated and visualized using GraphPad Prism 9.5 ( (Livak and Schmittgen, 2001; Wu et al., 2025).

## Results

### Identification, physicochemical properties, and secondary structure prediction of *AsbHLHs*

A combined study of genome-wide and full-length transcriptome-wide was carried out to screen and identify *bHLH* genes in *A. sinensis* via publicly available genomic sequences and our previously published full-length transcriptome data (GSA: CRA016571; https://ngdc.cncb.ac.cn/gsa). We identified a total of 148 *AsbHLH* family members based on the *A. sinensis* genome (**Supplementary Table 2**). To further characterize these identified *AsbHLHs*, we analyzed the physicochemical properties of the putative proteins. These 148 *As*bHLH proteins displayed a diversity in length, molecular weight, and theoretical isoelectric points (PIs). Results showed that the 148 *As*bHLH proteins’ length ranged from 83 to 743 amino acid residues, with a relative molecular weight and PIs fluctuating from 9.6 kDa to 80.8 kDa, and 4.55 to 9.81, respectively (**Supplementary Table 3**). Notably, the gene *AS10G02152* encoded the largest *As*bHLH protein (743 aa, 80.8 kDa), while the AS06G02346 encoded the smallest (83 aa, 9.6 kDa). Furthermore, the fat coefficient of these proteins ranged from 53.21 to 107.95, signifying diverse lipid interaction potentials (**Supplementary Table 3**). The average PI of 148 *As*bHLH proteins is about 6.7, ranging from a minimum PI of 4.5 (*AS09G01988*) to a maximum of 9.81 (*AS11G00848*). Among these, 47 bHLH proteins were basic (pI > 7.0), while the remaining were neutral or acidic. Additional analysis revealed that the average hydrophobicity index of all bHLH proteins is -0.56, indicating that the family inclusively has significant hydrophilic characteristics, with no hydrophobic protein members detected. The only *As*bHLH proteins include AS03G00994, AS06G02317, AS02G00486, AS08G00915, AS06G03884, and AS06G03247 are stably present, while the other family members have an instability index of more than 40. The subcellular localization prediction of the 148 *AsbHLHs* revealed that 147 *As*bHLH proteins are localized in the nucleus, while only one protein, AS07G00356, is located in the cytoplasm, indicating that these proteins have significantly nuclear localization characteristics. Moreover, an extrapolative analysis of the structural configuration of the Angelica bHLH family members was conducted. The results specified that the compositional ratio of the secondary structure of each Angelica bHLH protein differs dynamically, with the proportion of β-turns and extended chains being lower than that of random coils and α-helices (**Supplementary Table 3**).

### Phylogenetic relationship between bHLH proteins of *A. sinensis* and *Arabidopsis thaliana*

**Figure 1:**
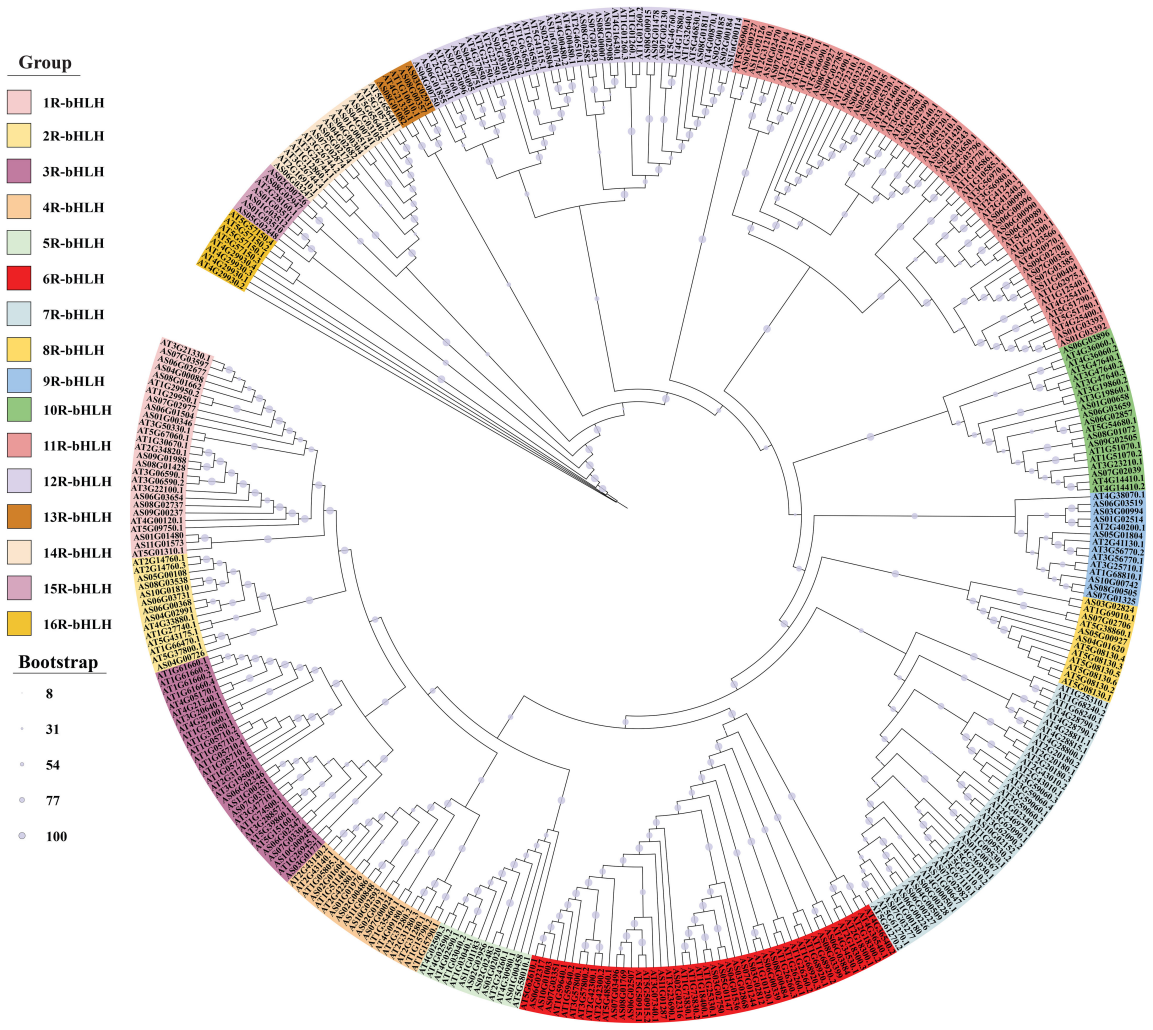
Comparison of the phylogenetic tree of bHLH proteins between *A. sinensis* and *A. thaliana*.

To classify the *A. sinensis* bHLH protein subfamilies and recognize the evolutionary relationships among the bHLH proteins from *A. sinensis* and *A. thaliana*, a phylogenetic tree was built using the sequences of the 148 *As*bHLH proteins and 225 A*. thaliana* bHLH proteins. Results indicated that the 148 *As*bHLH proteins were clustered into 16 subfamilies (1R- to 16R-bHLH) according to the tree topology and classification of the bHLH superfamily in *A. thaliana* (**Figure 1**). The subfamilies showed the following gene distribution: 1R-bHLH (n=14), 2R-bHLH to 4R-bHLH, 9R-bHLH and 10R-bHLH (7 for each), 5R-bHLH (5), 6R-bHLH (18), 7R-bHLH (10), 8R-bHLH (4), 11R-bHLH (26), 12R-bHLH (19), 13R-bHLH (3), 14R-bHLH (8) and 15R-bHLH (6). There is no 16R-bHLH subfamily, suggesting that Angelica bHLHs closely related to Arabidopsis bHLH proteins may share similar functions. Among these groups, the 11R-bHLH subfamily was the most abundant with 26 gene members (39% of total bHLHs), followed by 12R-bHLH containing 19 proteins (28%), while 13R-bHLH contains only 3 bHLH proteins (4.4%). The small subfamilies (e.g., 13R-bHLH with 3 members and 5R-bHLH with 5 members) propose potential evolutionary specialization or conserved functions between *A. sinensis* and *A. thaliana*. This phylogenetic classification permits functional prediction of *As*bHLHs based on characterized *At*bHLH proteins within the same subgroups, mainly for processes like secondary metabolite biosynthesis and stress responses, though experimental validation remains necessary.

### Gene structure and conserved motif analysis

**Figure 2:**
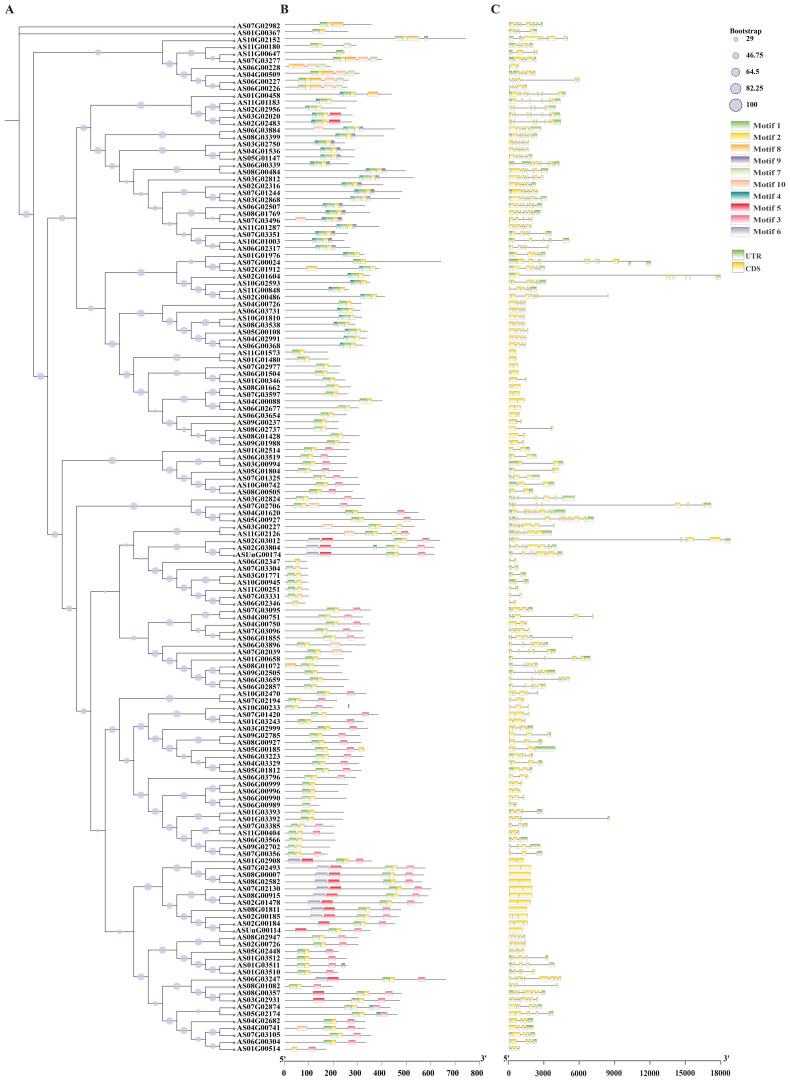
Evolutionary relationship, conserved motifs and genetic structures of *bHLH* genes in *A. sinensis*. **(A)** bHLH protein’s evolutionary relationship in *A. sinensis*; **(B)** Conserved motifs of *bHLH* genes in *A. sinensis*; **(C)***bHLH* genes structure in *A. sinensis*.

Phylogenetic analysis revealed that closely related bHLH members share similar motif compositions, demonstrating structural conservation (**Figure 2A**). MEME analysis identified 10 conserved motifs for bHLH proteins; motifs 1–5 were most prevalent, while motif 5 localized to the N-terminus and motifs 6/10 to the C-terminus (**Supplementary Figure 1**; **Figure 2B**). Gene structure analysis showed exon numbers ranging from 1 to 18 among the 148 *bHLH* genes, with 3–9 exons being most common; notably, *AS02G01604* and *AS02G03012* contained 18 exons and *AS07G02706* contained 16 exons, while two genes, *AS11G01573* and *AS01G01480*, possessed only one exon, suggesting potential functional diversification correlated with structural variation (**Figure 2C**).

### Conserved domain analysis

**Figure 3:**
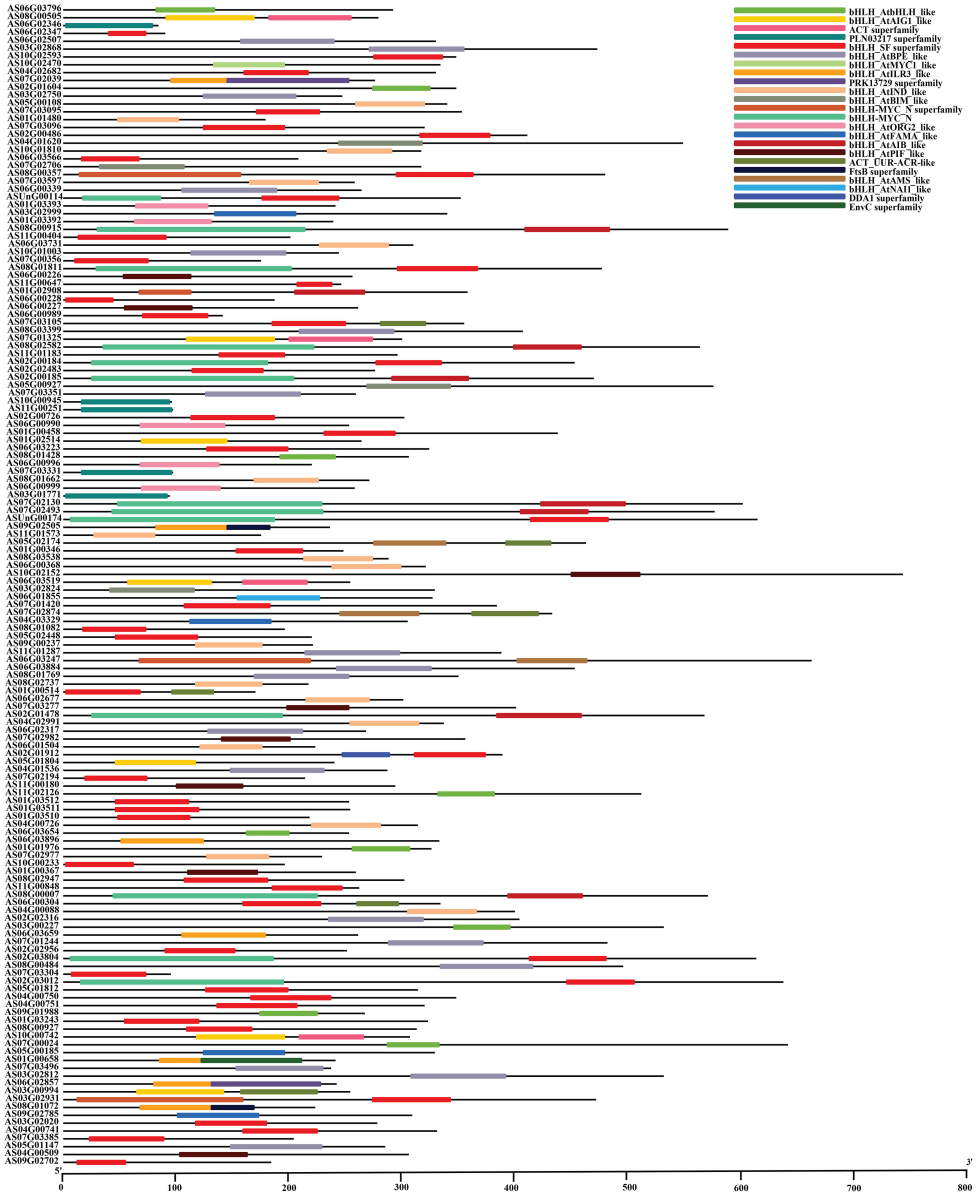
Conserved structural domains of bHLH proteins in *A. sinensis*.

**Figure 4:**
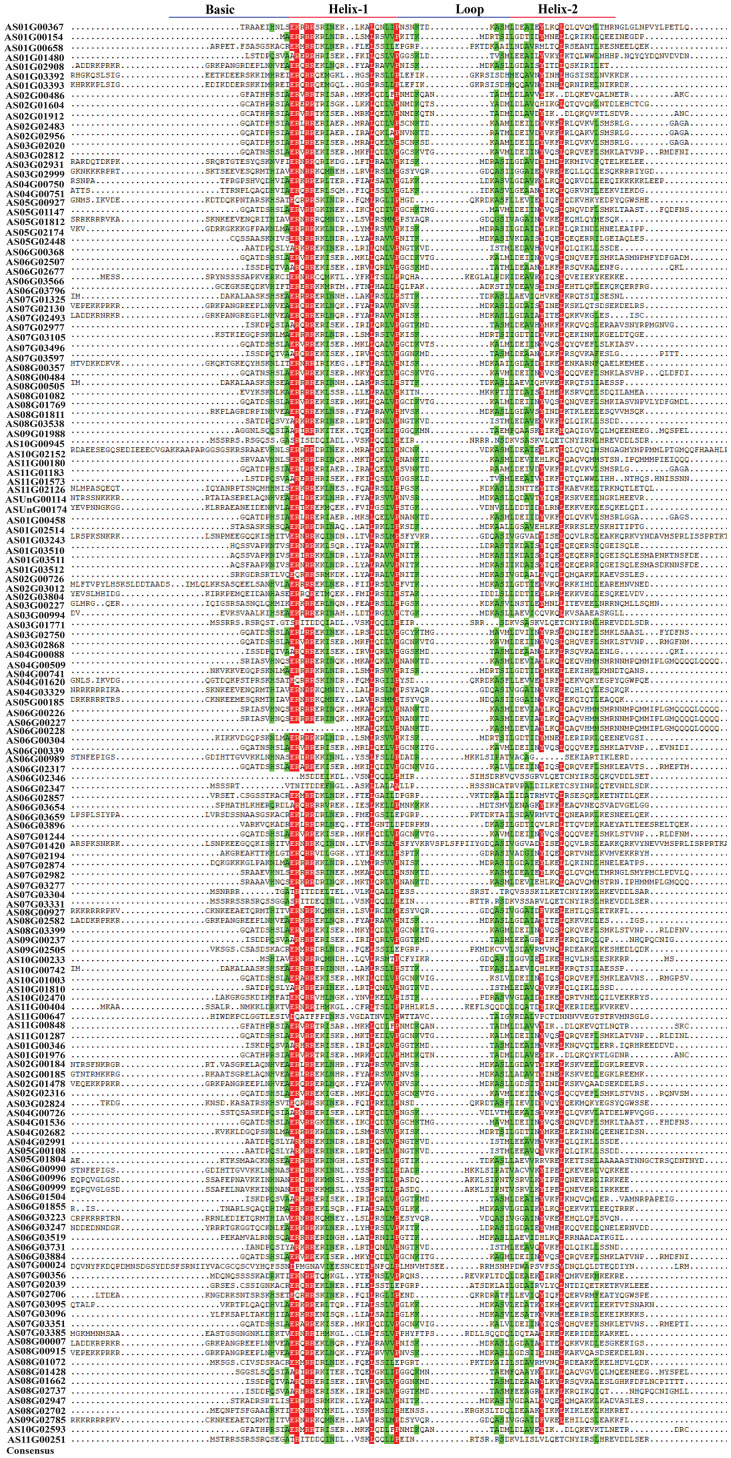
Multiple alignment of conserved domain amino acid sequences of multiple bHLH proteins from *A. sinensis*.

The angelica bHLH proteins encompass a total of 23 distinct domain types (**Figure 3**). These 148 bHLH amino acid sequences contain 7 superfamilies, including PLN03217 superfamily, bHLH_SF superfamily, PRK13729 superfamily, bHLH_MYC_N superfamily, FtsB superfamily, DDA1 superfamily and EnvC superfamily. The amino acid sequences of 5 genes named *AS06G02346*, *AS10G00945*, *AS11G00251*, *AS07G03331* and *AS03G01771* belong to PLN03217 superfamily while only 4 genes *AS08G00357*, *AS01G02908*, *AS06G03247* and *AS03G02931*, belong to bHLH_MYC_N superfamily. Other than superfamilies, the domain bHLH_MYC_N contains 13 gene sequences including *ASUnG00114*, *AS08G00915*, *AS08G01811*, *AS08G02582*, *AS02G00184*, *AS02G00185*, *AS07G02130*, *AS07G02493*, *ASUnG00174*, *AS02G01478*, *AS08G00007*, *AS02G03804*, and *AS02G03012*. Consequently, the multiple sequence alignment of the bHLH domain sequence of 148 *AsbHLHs* revealed that the basic region and two helixes were highly conserved in most of *As*bHLH proteins, except that the basic region was absent in AS01G00154, AS06G00228, and AS06G02346 (**Figure 4**).

### Chromosomal localization and synteny analysis

**Figure 5:**
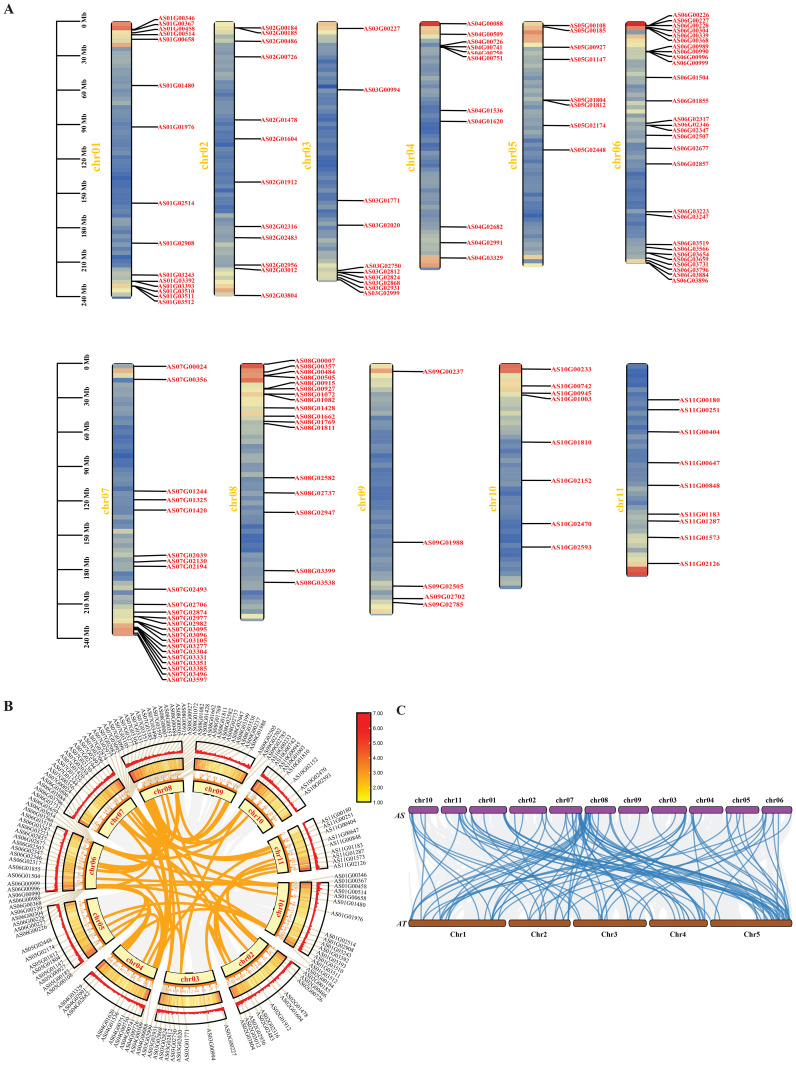
Chromosomal localization and synteny analysis. **(A)** Chromosomal distribution and regional duplication of 148 *bHLH* genes of *A. sinensis*. The scale bar on the left indicates the length (Mb) of chromosomes; **(B)** The collinearity circle diagram of the *A. sinensis* genome; **(C)** Collinearity analysis among the genomes of *A. sinensis* and *A. thaliana*; the blue line represents the *bHLH* gene pairs..

The results of the chromosomal localization of Angelica *bHLH* genes indicate that 148 Angelica *bHLH* genes are distributed on 11 chromosomes, with the highest number of *bHLH* genes (28) located on chr06 and the lowest (5) on chr09 (**Figure 5A**). Within the Angelica *bHLH* transcription factor family, there are 64 pairs of genes that have syntenic relationships (**Figure 5B**). Chromosomes chr01 to chr08 exhibit synteny relationships with *bHLH* genes located on chr02 to chr11, with chr02, chr03, and chr04 showing more distinct syntenic relationships with other chromosomes. Consequently, the synteny analysis of the *AsbHLH* and *AtbHLH* genomes reveals that there is a significant synteny relationship between the *bHLH* family members in *A. sinensis* and those in *A. thaliana*, with most of the genes on chromosomes 7, 8, and 11 of *A. sinensis* corresponding to genes on chromosomes 1 and 5 of *A. thaliana* (**Figure 5C**).

### Cis-acting elements in the promoter region of the *AsbHLHs*

**Figure 6:**
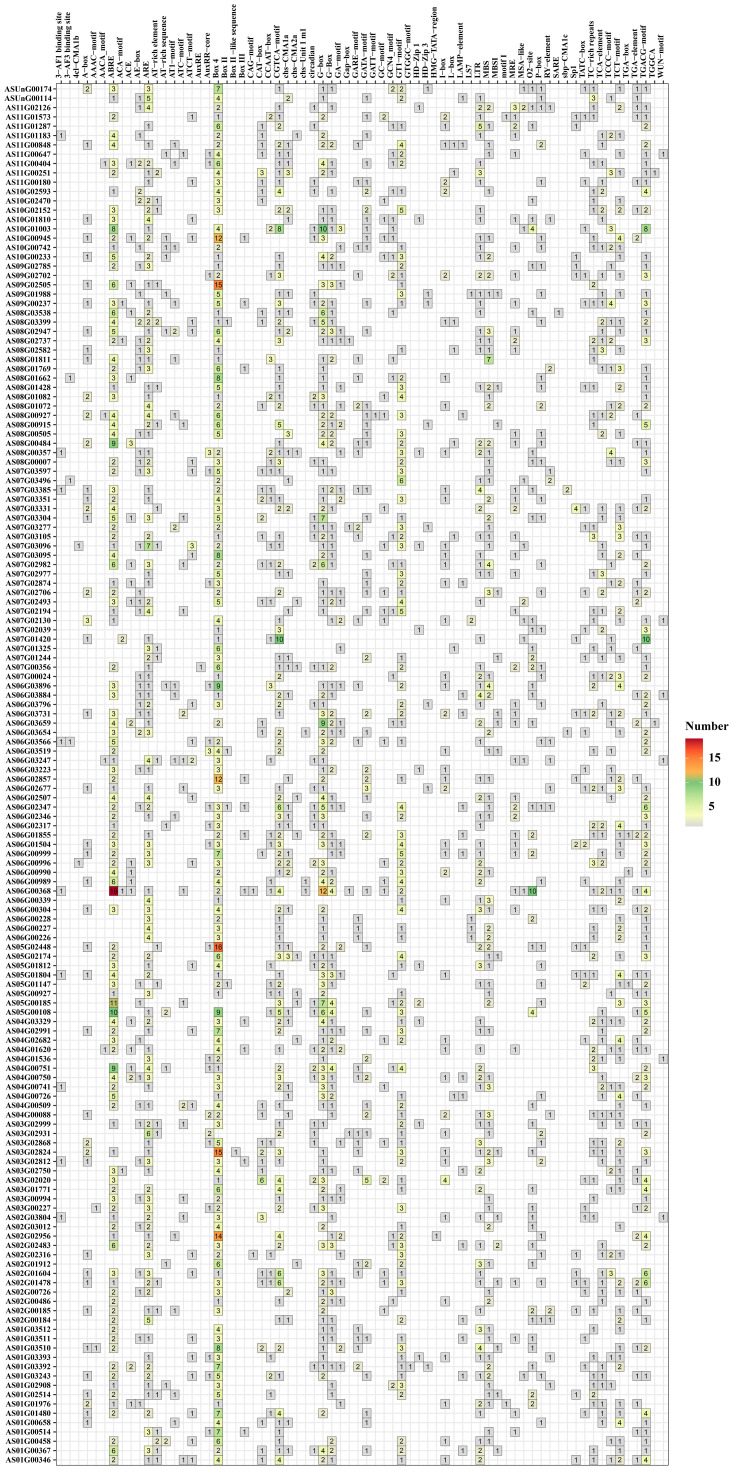
Distribution of cis-acting elements in the *bHLH* gene promoters of *A. sinensis*.

The upstream 2000 bp promoter region of the angelica *bHLH* gene sequence predicted 57 types of cis-acting elements and it was found that most of the angelica *bHLH* gene promoter regions contain hormone response elements, including abscisic acid, methyl jasmonate (MeJA), jasmonic acid (JA), auxin (AU), salicylic acid (SA), and gibberellin (GA) (**Figure 6**). Additionally, the majority of the angelica *bHLH* promoter regions also have light-responsive elements (Box 4, G-Box, GT1 motif, ATCT motif, AAAC motif, etc.), defense and stress response elements (LTR and TC-rich repeats), drought-induced *bHLH* binding sites (MBS), and cis-regulatory elements necessary for anaerobic induction (ARE), among other cis-acting elements. A small number contain bHLH binding sites (AF) involved in the regulation of flavonoid biosynthesis and wound response elements (WUN motif). It was found that although the angelica *bHLH* genes within the same group have close relationships, there are significant differences in the types and quantities of hormone response elements in their promoter regions, indicating that the expression regulatory mechanisms of the angelica *bHLH* family members exhibit specificity and complexity.

### Identification of co-expression modules by WGCNA

**Figure 7:**
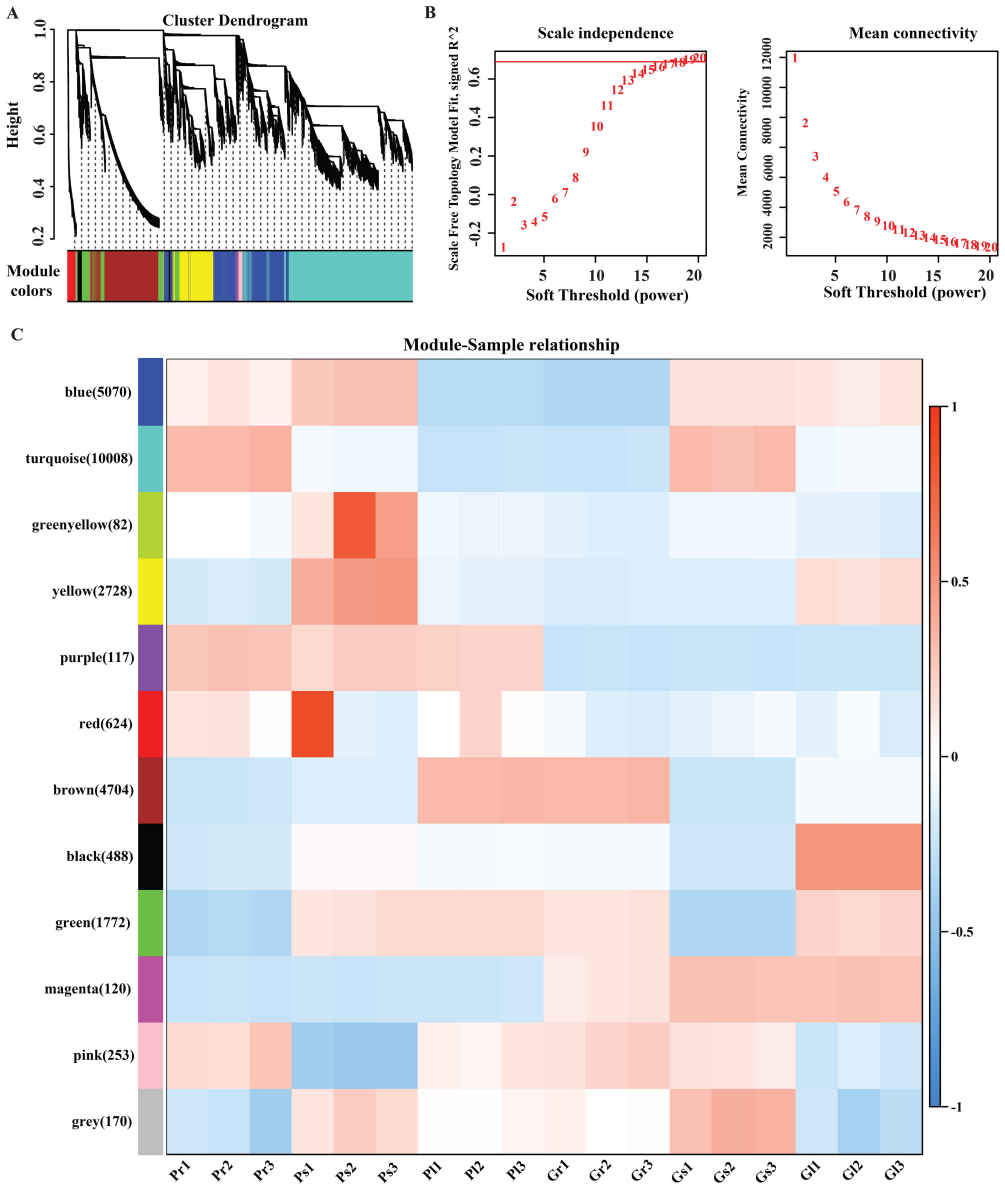
**(A)** Dendrogram of all expressed modules (each color represents a different module); **(B)** Soft threshold and mean connectivity; **(C)** Module trait correlation. The color key, ranging from blue to red, represents R2 values from -1 to 1.

To attain an inclusive understanding of genes expressed in the different tissues of *A. sinensis* and to identify the specific genes that are highly associated with ferulic acid biosynthesis in different plant tissue samples, WGCNA was performed (Langfelder and Horvath, 2008). To increase the accuracy of WGCNA, we used our previously published expression data of 18 samples (**Supplementary Table 4**) (Arshad et al., 2024). After filtering out the genes with a small coefficient of variation, 26,137 genes were selected for the WGCNA analysis, resulted in highly correlated gene clusters known as modules. Specifically, we identified 12 distinct modules of highly interconnected genes, shown in the dendrogram (**Figure 7A**), in which the major tree branches define the modules, based on soft threshold and mean connectivity (**Figure 7B**). Each module correlated with distinct samples due to sample-specific expression profiles (**Supplementary Table 5**). Gene co-expression modules showed distinct tissue specificity, with the turquoise module (10,008 genes) correlated with stems and the blue module (5,070 genes) with leaves (**Figure 7C**). The turquoise module and blue module were notably enriched for 32 and 8 *bHLH* transcription factors, and for three and one phenylpropanoid biosynthesis pathway-specific genes, respectively, associated with ferulic acid biosynthesis.

### Expression analysis of the *AsbHLH* genes and protein-protein interaction

**Figure 8:**
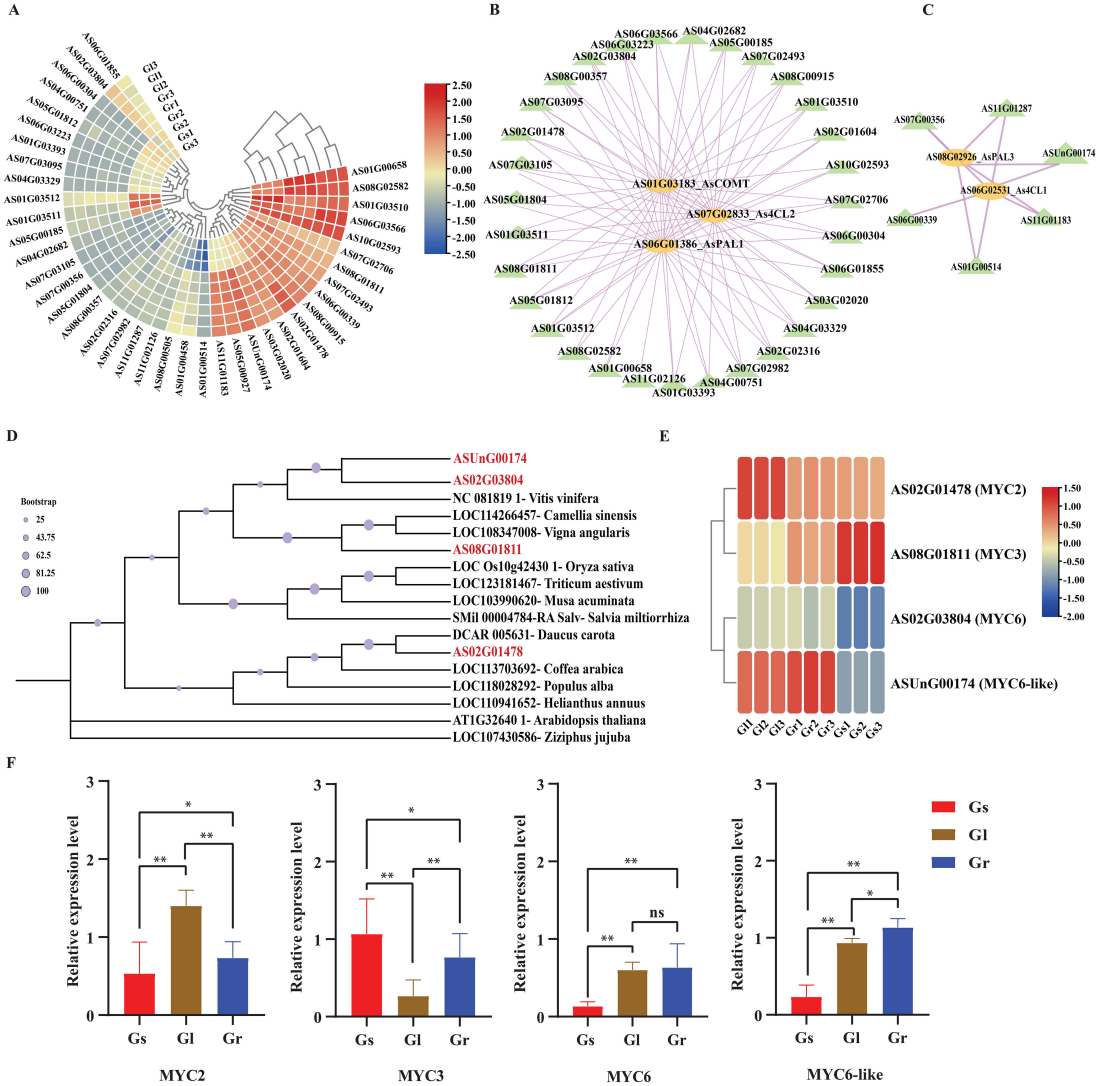
Identification of candidate *bHLH* genes regarding ferulic acid regulation. **(A)** Expression profiles analysis of *bHLH* genes in *A. sinensis*; Gr: Green root, Gl: Green leaf, and Gs: Green stem. Different colors in the figure represented different log2 (FPKM); **(B)** Protein-protein interaction among *bHLH* genes and phenylpropanoid pathway-specific genes of turquoise module; **(C)** Protein-protein interaction among *bHLH* genes and phenylpropanoid pathway-specific genes of blue module; **(D) ** phylogenetic tree of *AsbHLH* in *A. sinensis* and *bHLHs* in other species; **(E)** heatmap presenting expression levels of candidate genes in three tissues; **(F)** qRT-PCR results of four candidate genes in three tissues.

The identified *AsbHLH* genes from turquoise and blue modules had differential expression levels, as determined by pairwise comparative analysis of each plant tissue sample (**Supplementary Table 6**). A heatmap of these identified *AsbHLH* genes from two modules was created by using their expression levels from three different tissues (leaf, stem and root). The results indicated that these *AsbHLH* genes have approximately the same expression levels in leaf and root samples as compared to the stem (**Figure 8A**). Therefore, using all 32 and 8 genes, we constructed a protein-protein interaction (PPI) network incorporating phenylpropanoid biosynthesis pathway-specific genes (responsible for ferulic acid biosynthesis) based on Spearman correlation coefficient (R > 0.8) and p-value < 0.05 (**Supplementary Figure 2**; **Figures 8B, C**). Results showed that the 8 *AsbHLH* genes in turquoise module, including, *AS02G03804* (*MYC6*), *AS06G01855* (*bHLH-25*), *AS02G01478* (*MYC2*), *AS08G01811* (*MYC3*), *AS06G03566* (*bHLH-162*), *AS01G03510* (*bHLH-35*), *AS07G02982* (*bHLH-24*), and *AS02G02316* (*bHLH-62*), were highly correlated to the pathway-specific enzymes *AsPAL1*, *As4CL2* and *AsCOMT*. Similarly, in the blue module, 3 genes, including *ASUnG00174* (*MYC6*-like), *AS11G01183* (*bHLH-59*) and *AS11G01287* (*bHLH-62*) were highly correlated with the core enzymes *AsPAL3* and *As4CL1* of the phenylpropanoid biosynthesis pathway, which are associated with the ferulic acid biosynthesis.

### Screening of putative *AsbHLH* genes regulating ferulic acid biosynthesis, and qRT-PCR verification

By selecting bHLH proteins known to be associated with regulating ferulic acid biosynthesis and performing homologous comparison and systematic analysis with bHLH proteins in Angelica, a total of 4 homologous *As*bHLH proteins were screened from the genomic data of Angelica, which are potential homologs to known functional proteins (*Vitis vinifera*, *Arabidopsis thaliana*, *Camellia sinensis*, *Oryza sativa*, *Triticum aestivum*, *Vigna angularis*, *Musa acuminata*, *Salvia miltiorrhiza*, *Daucus carota*, *Coffea arabica*, *Populus alba*, *Helianthus annuus*, and *Ziziphus jujuba*) (**Figure 8D**). These 4 Angelica *bHLH* genes may be the potential genes for future research on ferulic acid biosynthesis. An expression pattern analysis was conducted on 4 putative genes with good expression reproducibility, revealing that *AsMYC2* and *AsMYC3* have stable expression levels in all three tissues as compared to *AsMYC6* and *AsMYC6*-like (**Figure 8E**). Based on homologous comparison and systematic analysis, these 4 *AsbHLH* genes, homologous to those known to be associated with ferulic acid biosynthesis, were selected for quantitative fluorescent determination. Primers were designed using Primer3 (**Supplementary Table 1**). The results of these putative genes indicated that *AsMYC2* presented a relatively higher expression level in leaves than in roots. At the same time, *AsMYC3* and *AsMYC6* had relatively lower expression levels in leaves compared to roots. Consequently, the *AsMYC6*-like represented a higher expression level as compared to all others and *AsMYC6* did not show significant differences in expression levels between leaves and roots, which is consistent with transcriptome data (**Figure 8F**).

## Discussion

Understanding the molecular mechanism that controls ferulic acid biosynthesis in A*. sinensis* is crucial for enhancing its therapeutic value. Ferulic acid biosynthesis is tightly regulated and highly coordinated by the phenylpropanoid pathway (Gao et al., 2023). Several studies reported that *bHLH* genes are important regulators of secondary metabolite production in plants (Zhou and Memelink, 2016; Wang et al., 2018, 2025; Guo et al., 2021). Therefore, in this study, we performed a genome-wide association study of the *bHLH* gene family in *A. sinensis* to find the putative genes potentially regulating ferulic acid production. The results provide comprehensive information on gene correlations involved in the regulation of ferulic acid biosynthesis. This study also shows that PPI is quite helpful in measuring the correlation between genes and transcription factors.

### Genome-wide identification and evolutionary analysis of *bHLH* transcription factors in *A. sinensis*

bHLH is one of the biggest transcription factor families in plants (Sun et al., 2018; Wang et al., 2018; Guo et al., 2021; Hao et al., 2021). Based on the genomic data of *Angelica sinensis*, this study annotated 148 *bHLH* transcription factors, which is similar to the distribution of *bHLH* genes in *Salvia miltiorrhiza* (151 genes) (Zhang et al., 2015), *Daucus carota* (146 genes) (Chen et al., 2015), *Oryza sativa* (167 genes) (Li et al., 2006) and tomato (152 genes) (Wang et al., 2015). It is much lower than the *Triticum aestivum* (571 *bHLH* genes) (Wei and Chen, 2018), higher than the *Cannabis sativa* (99 *bHLH* genes) (Bassolino et al., 2020), and much higher than the *Cyanidioschyzon merolae* (1 *bHLH* genes) (Pires and Dolan, 2010). This may be related to plant evolution and development. The *bHLH* family genes were divided into 21 subfamilies in *A. thaliana* (Toledo-Ortiz et al., 2003), 23 in *M. domestica* (Yang et al., 2017), 17 in *Ginkgo biloba* (Zhou et al., 2020) and 19 in peach (Zhang et al., 2018). In this study, the evolutionary analysis identified 148 *bHLH* genes in *A. sinensis*, which were divided into 16 subfamilies (**Supplementary Table 2**; **Figure 1**). Our results on *AsbHLHs* showed similarities as well as differences compared to the classifications of *A. thaliana*. In general, the structures and functions of *AsbHLH* matched those of *A. thaliana*. These results indicate that certain *bHLHs* play strongly conserved roles in *A. sinensis* and *A. thaliana*, whereas other *bHLHs* may have specific functions. Consequently, the conserved motifs are crucial for proteins to perform biological functions. The results of conserved motif analysis of *A. sinensis bHLHs* showed that motifs 1–5 appeared frequently (**Figure 2**). bHLH members of the same subfamily share similar motif compositions. However, a few bHLH proteins clustered within a single subfamily exhibited distinct motif compositions, suggesting that this may be related to the functional differentiation of *A. sinensis bHLH* family members (**Figure 2**). Different subfamily members also contain distinct or specific motifs. These results suggest that the N-terminus of bHLH transcription factors is not completely conserved among different subfamily members, implying that these motifs may have different biological functions in *A. sinensis*. Furthermore, these *AsbHLHs* possess a highly conserved domain HLH at the C-terminus, which binds to the G-box cis-element in the MEJA and JA-responsive promoters (**Figures 3**, **4**) (Fernández-Calvo et al., 2011; Figuora 2012). Further research into the functions and regulatory mechanisms of different motifs will provide a more comprehensive understanding of the functions and regulatory networks of bHLH transcription factors.

### Identification and regulatory role of *AsbHLH* genes in ferulic acid biosynthesis

Taken together, the evolutionary analysis results and *bHLH* genes with known functions can be combined to predict the *AsbHLH* genes related to growth and development, secondary metabolism, and environmental responses in *A. sinensis*. The regulation of growth and development, stress resistance, and signal transduction by bHLH transcription factors has been reported in several plants (Zhou and Memelink, 2016; Sun et al., 2018; Wang et al., 2018, 2025; Mathesius, 2020; Guo et al., 2021). To date, at least 43 *bHLH* transcription factors have been identified as regulators of secondary metabolism in at least 21 distinct plants, and these active components include flavonoids, anthocyanins, alkaloids, tanshinone, and terpenoids (Zhang et al., 2015). *bHLH* transcription factor genes are expressed in stimulus-responsive, constitutive, or organ-specific manners. In addition, available functional information from *Zea mays* (Zhang et al., 2018), *A. thaliana* (Zhao et al., 2019), and *Matthiola incana* (Nuraini et al., 2020) suggests that *bHLH* transcription factors can regulate the phenylpropanoid biosynthesis pathway in both a positive and negative fashion.

The phenylpropanoid pathway is crucial for producing lignin (structural support), flavonoids (UV protection, pigments), ferulic acid and defense compounds (phytoalexins, antioxidants) (Dixon et al., 2002; Sharma et al., 2019; Yadav et al., 2020). Underlying this pathway, the *Caffeic acid O-methyltransferase* (COMT) is a key enzyme responsible for ferulic acid production (Eckardt, 2002; Narnoliya et al., 2021; Liang et al., 2022; Yang Y. et al., 2023). Previous studies have demonstrated that *COMT* catalyzes the methylation of caffeic acid to ferulic acid, a rate-limiting step in the pathway (Narnoliya et al., 2021; Shang et al., 2025). Our findings align with these reports, as we observed that *COMT* expression strongly correlates with ferulic acid accumulation in *A. sinensis*. Furthermore, phylogenetic, promoter analysis and protein-protein interactions of the identified genes in the turquoise module suggested that *AsMYC2*, *AsMYC3*, *AsMYC6*, and *AsMYC6*-like genes are highly correlated and potentially directly regulate *COMT*, implying a mechanistic link between these transcription factors and ferulic acid biosynthesis (**Figure 8**). This potential regulatory relationship is consistent with studies in other plants, where *bHLH* transcription factors have been shown to bind to the promoters of phenylpropanoid biosynthetic genes (Zhou and Memelink, 2016; Narnoliya et al., 2021; Shang et al., 2025).

Among the bHLH members, *MYC* genes have been extensively studied for their regulatory role in secondary metabolite biosynthesis. For instance, in *A. thaliana*, *MYC2* is a well-characterized transcription factor that modulates jasmonate signaling and influences phenylpropanoid metabolism (Dombrecht et al., 2007; Kazan and Manners, 2013). In another study in *A. thaliana* reported the role of *MYC3*, along with *MYC2* and *MYC4*, was reported in regulating JA-mediated defense responses (Dombrecht et al., 2007; Cheng et al., 2011; Fernández-Calvo et al., 2011; Wang et al., 2017). Similarly, in *Medicago truncatula*, *MYC2* has been shown to regulate flavonoid biosynthesis by binding to the promoters of key biosynthetic genes (Zhou and Memelink, 2016; Mathesius, 2020; Naik et al., 2021). In *Solanum lycopersicum*, *SIMYC3* regulates jasmonic acid biosynthesis (Wang et al., 2023). Consequently, in *Catharanthus roseus* (L.) G. Don, the *bHLH* transcription factor *CrMYC2* regulates *ORCA* gene expression, which in turn regulates alkaloid biosynthesis genes (Colinas et al., 2021). Furthermore, in *Salvia miltiorrhiza*, *SmMYC2* regulates tanshinone biosynthesis (Shi et al., 2020). Meanwhile, in rice and tomato, *MYC1* and *MYC6* (orthologs of *AtMYC3/4*) are involved in drought and JA signaling (Fu et al., 2017; Feng et al., 2023). Overall, in *A. thaliana* and other plants, *MYC6* is less studied compared to *MYC2*/*MYC3* but is implicated in JA-mediated stress responses and may have redundant or complementary roles with other *MYC* TFs. These findings highlight the conserved role of *MYC* genes in phenylpropanoid regulation across different plant species. In our study, we identified *AsMYC2* and *AsMYC3* as homologs of *AtMYC3*, suggesting their potential involvement in the phenylpropanoid pathway, particularly in ferulic acid biosynthesis. Further, the fluorescence quantitative results of the four selected *AsbHLHs* in this study were consistent with the transcriptome data (**Figure 8D**). Through comparative studies of MYC homologs across multiple plant species, our study suggests that *AsMYC2*, *AsMYC3*, *AsMYC6*, and *AsMYC6*-like may regulate ferulic acid accumulation in roots, leaves, and stems of *A. sinensis*. However, further functional characterization is required to confirm their direct binding to the *COMT* promoter, as well as it is vital to clearly state that the functional roles projected in this study for the identified *AsbHLH* genes, chiefly their regulatory influence on COMT and ferulic acid biosynthesis, are currently hypothetical and based on in silico analyses and correlation data. Conclusively, this study establishes a critical theoretical framework for understanding the regulatory network of *bHLH* transcription factors in ferulic acid biosynthesis in *A. sinensis*, providing a strong foundation for future gene functional studies and medicinal quality research.

## Conclusion

This study systematically identified 148 *bHLH* transcription factors using whole-genome data from *Angelica sinensis*. Our findings demonstrate that *AsMYC2*, *AsMYC3*, *AsMYC6*, and *AsMYC6*-like genes are potential regulators of ferulic acid biosynthesis in *A. sinensis*, likely through their interaction with the *COMT* gene. This hypothesis is further supported by qRT-PCR analysis. These results strengthen the evidence that *bHLH* transcription factors play a central role in ferulic acid production in this medicinal plant. This study advances our understanding of the regulatory mechanisms governing ferulic acid biosynthesis. The identified genes and their potential *COMT* regulatory axis provide valuable tools for future research, enabling both functional validation and exploration of their applications for enhanced ferulic acid production in *A. sinensis* through genetic engineering or breeding approaches.

## Data Availability

The original contributions presented in the study are included in the article/**Supplementary Material**. Further inquiries can be directed to the corresponding author.

## Supplementary material

Supplementary Figure 1 Meme predicted 10 motifs for the AsbHLH transcription factors.

Supplementary Figure 2 Heatmap visualization and phenylpropanoid biosynthesis pathway. **(A)** Expression profile analysis of Phenylpropanoid biosynthesis pathway genes in *A. sinensis*; Gr: Green root, Gl: Green leaf, and Gs: Green stem. Different colors in the figure represented different log2 (FPKM); **(B)** Phenylpropanoid biosynthesis pathway.
